# High-Temperature Nano-Indentation Creep of Reduced Activity High Entropy Alloys Based on 4-5-6 Elemental Palette

**DOI:** 10.3390/e22020230

**Published:** 2020-02-18

**Authors:** Maryam Sadeghilaridjani, Saideep Muskeri, Mayur Pole, Sundeep Mukherjee

**Affiliations:** Department of Materials Science and Engineering, University of North Texas, Denton, TX 76203, USA; Maryam.Sadeghilaridjani@unt.edu (M.S.); saideepmuskeri@my.unt.edu (S.M.); mayurpole@my.unt.edu (M.P.)

**Keywords:** refractory high entropy alloys, creep, nano-indentation, stress exponent, activation energy, activation volume

## Abstract

There is a strong demand for materials with inherently high creep resistance in the harsh environment of next-generation nuclear reactors. High entropy alloys have drawn intense attention in this regard due to their excellent elevated temperature properties and irradiation resistance. Here, the time-dependent plastic deformation behavior of two refractory high entropy alloys was investigated, namely HfTaTiVZr and TaTiVWZr. These alloys are based on reduced activity metals from the 4-5-6 elemental palette that would allow easy post-service recycling after use in nuclear reactors. The creep behavior was investigated using nano-indentation over the temperature range of 298 K to 573 K under static and dynamic loads up to 5 N. Creep stress exponent for HfTaTiVZr and TaTiVWZr was found to be in the range of 20–140 and the activation volume was ~16–20*b*^3^, indicating dislocation dominated mechanism. The stress exponent increased with increasing indentation depth due to a higher density of dislocations and their entanglement at larger depth and the exponent decreased with increasing temperature due to thermally activated dislocations. Smaller creep displacement and higher activation energy for the two high entropy alloys indicate superior creep resistance compared to refractory pure metals like tungsten.

## 1. Introduction

There is a strong demand for refractory metals for use in the harsh environment of next-generation nuclear reactors because of their high melting point, elevated temperature strength, and creep resistance [1,2]. High entropy alloys (HEAs) or complex concentrated alloys (CCAs), composed of multiple principal elements in equimolar or near equimolar proportions, have drawn intense attention as structural materials for the nuclear industry due to their excellent elevated temperature properties and irradiation resistance [3,4,5]. These systems consist of a single phase in HEAs or multiple solid solution phases in CCAs [3,4]. In particular, refractory high entropy alloys (R-HEAs) constituting refractory elements promise a gamut of excellent elevated temperature properties [6,7,8]. HfTaTiVZr and TaTiVWZr are two newly developed HEAs consisting of 4-5-6 refractory elements [5,9]. These alloys were found to have higher strength at elevated temperatures, superior to conventional nuclear reactor materials such as P92 and SS316 steels [9]. The inherently low activity of the constituent elements makes them attractive for next-generation nuclear reactor applications due to the short “hands-on” time and easier post-service recycling prospect [9].

In service, one of the essential design criteria for engineering components is the time-dependent deformation behavior (i.e., creep resistance). Macroscopic bulk compression or tension [10,11,12], micro-pillar compression [13,14], and nano-indentation technique [15,16,17,18,19,20,21] have been used to investigate the deformation behavior of materials in a wide range of length scales. Major discrepancies between the data obtained by nano-indentation and via conventional creep testing (i.e., compression/tension) have been reported [22,23]. This may be due to the complex multi-axial and inhomogeneous stress below indenters in comparison with uniaxial and homogeneous stress during bulk compression/tension test [24]. Despite these discrepancies, nano-indentation has been used as a convenient and fast approach to characterize the time-dependent deformation behavior at the small-scale for pure metals [16,17], bulk metallic glasses [25,26], and HEAs [15,18,27,28,29,30,31,32]. Most nano-indentation creep studies are limited to room temperature, low load (<100 mN), and face-centered cubic (FCC) metals/alloys [15,27,28,29,30,31,32]. However, there is a very limited understanding of local deformation processes in body-centered cubic (BCC) HEAs, more specifically for the newly developed refractory high entropy alloys at high load (>1 N) and elevated temperatures.

Here, the nano-indentation creep behavior was evaluated for two novel refractory high entropy alloys, HfTaTiVZr and TaTiVWZr, at high load and elevated temperature. We performed two sets of experiments in the current study: (i) static constant load hold (CLH) protocol was adopted for one set of experiments to exclude possible surface effects and (ii) dynamic mechanical analysis (DMA) mode was used to study oscillatory load and indentation size effect. This allowed the comparison of response from static and dynamic loads, for which there is no systematic study in literature. The effect of temperature on the creep behavior of the R-HEAs was also investigated systematically. Stress exponent, activation volume, and activation energy of alloys at different combinations of temperature and loads were compared with pure tungsten (W), a reference metal used in nuclear reactors [33].

## 2. Experimental

Alloys with a nominal composition of HfTaTiVZr (named as Ta-Hf alloy hereafter) and TaTiVWZr (named as Ta-W alloy hereafter) with an equimolar proportion of constituent elements were prepared by arc-melting high purity elements (>99.9%) in a Ti-gettered argon atmosphere. The ingots were flipped and remelted at least four times to ensure chemical homogeneity. The as-cast alloys and pure W were polished with silicon carbide papers and diamond suspension to a mirror finish for microstructural characterization and nano-mechanical tests. The structure of the alloys was characterized using the Rigaku III Ultima X-ray diffractometer (XRD Rigaku Corporation, Tokyo, Japan) with a 1.54 Å wavelength Cu-Kα radiation. Scanning electron microscopy (SEM) was done using FEI Quanta ESEM (FEI Company, Hillsboro, OR, USA) with in-built energy-dispersive spectroscopy (EDS) to analyze the grain size and microstructure. Transmission electron microscopy (TEM) of as-cast alloys was performed on FEI Tecnai F20 operating at 200  kV. Thin foils for transmission electron microscopy were made using FEI Nova NanoLab 200 focused ion beam SEM (FIB-SEM).

Nano-indentation creep tests were done using Triboindenter TI-Premier (Bruker, Minneapolis, MN, USA) equipped with the XSol600 heating stage to heat samples up to 600 °C. The tests were done in an Ar+5% H_2_ gas environment to avoid oxidation. A Berkovich sapphire tip was used for all the creep tests. A standard fused quartz reference sample was used for the initial tip calibration. For each material, two different types of tests were performed: (i) static constant load hold (CLH) and (ii) dynamic load hold in DMA mode. The static creep tests were done by ramping the load to 1 N and 5 N at temperatures of 298 K, 423 K, and 573 K, and then held at maximum load for 120 s to determine creep response before unloading. High loads were used to avoid surface effects. In dynamic load tests, 100 mN, 500 mN, and 1000 mN loads were used to study the creep behavior. The frequency and amplitude were set to 100 Hz and 10% of the peak load, respectively. In all tests, a high loading rate of 20 mN/s was chosen to minimize plastic deformation during the loading segment. The samples were held at a prescribed temperature for at least 20 min to reduce the temperature gradient between the tip and the sample and to allow the indenter tip to reach a steady state. In addition, thermal drift was automatically corrected by the Triboindenter software and was between 0.05 and 0.1 nm/s during testing. At least 12 indents were made for each condition to get statistical variation. The distance between two indents was kept larger than 100 μm to avoid overlapping of their plastic zones.

## 3. Results 

**Figure 1:**
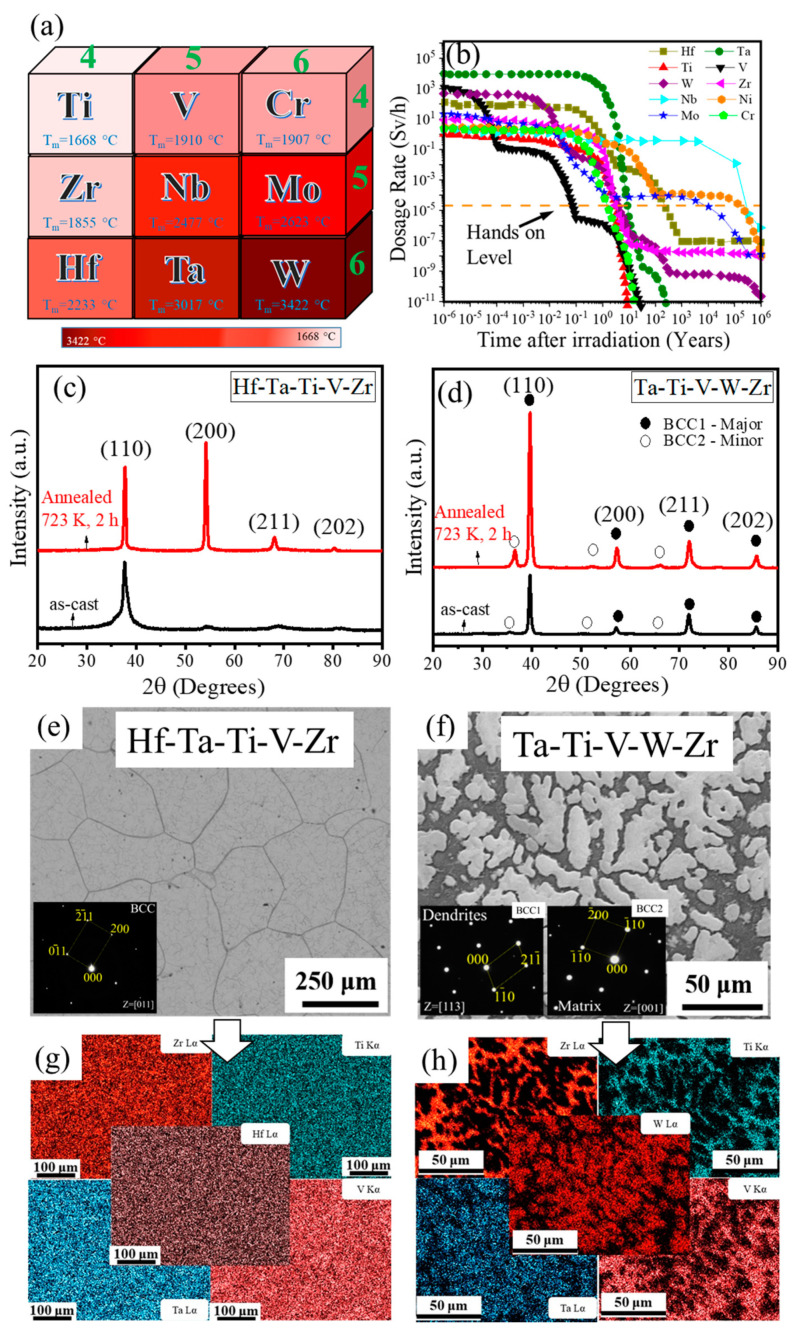
(**a**) Refractory elements belonging to the 4-5-6 group/period; (**b**) time in years required for group 4-5-6 refractory elements to reach “hands-on” level after exposure [34]. X-ray diffraction analysis of (**c**) HfTaTiVZr (Ta-Hf) and (**d**) TaTiVWZr (Ta-W) refractory high entropy alloys in as-cast and annealed conditions showing single-phase body-centered cubic (BCC) crystal structure for Ta-Hf and a BCC1 major phase and BCC2 minor phase for Ta-W; backscattered scanning electron microscopy image of (**e**) Ta-Hf and (**f**) Ta-W alloys showing equiaxed grains with an average grain size of ~250 μm for Ta-Hf and formation of two phases in Ta-W; insets showing selected area diffraction pattern of the alloys. Energy-dispersive X-ray spectroscopy of (**g**) Ta-Hf and (**h**) Ta-W alloys confirming a homogeneous distribution of elements in Ta-Hf alloy and partitioning of Ta and W into dendrite phase and Ti, V, and Zr into the matrix in Ta-W alloy.

All constituent elements of the two current refractory high entropy alloys belong to the 4-5-6 elemental palette with high melting points as shown in Figure 1a. The relatively short period of time required for the refractory elements found in the current HEAs (i.e., Hf, W, Ta, Ti, V, and Zr) compared to other elements such as Mo, Nb, and Ni to reach “hands-on” level after irradiation is shown in Figure 1b [34]. X-ray diffraction patterns of Ta-Hf and Ta-W HEAs in as-cast and annealed (at 723 K for 2 h) conditions are shown in Figure 1c,d, respectively. The peaks were indexed to a single phase BCC structure for Ta-Hf without any secondary phases or precipitates. Ta-W, on the other hand, showed a BCC1 major phase and a BCC2 minor phase. Both HEAs annealed at 723 K for 2 h showed identical structure as the cast alloy confirming their good microstructural stability. Backscattered SEM images of the as-cast Ta-Hf and Ta-W alloys are shown in Figure 1e,f, respectively. Insets show selected area diffraction (SAD) pattern along [011] zone axis for Ta-Hf alloy and along [113] and [001] zone axis for dendritic and inter-dendritic (matrix) phases of Ta-W alloy, respectively. Ta-Hf alloy showed equiaxed grains with an average grain size of ~250 μm. Corresponding EDS map of Ta-Hf alloy in Figure 1g confirms no obvious segregation and relatively homogeneous distribution of the constituent elements. On the contrary, SEM/EDS compositional analysis of Ta-W (Figure 1f,h) revealed two distinct phases, where Ta and W partitioned to the bright dendritic phase, while the darker matrix was rich in Ti, V, and Zr.

**Figure 2:**
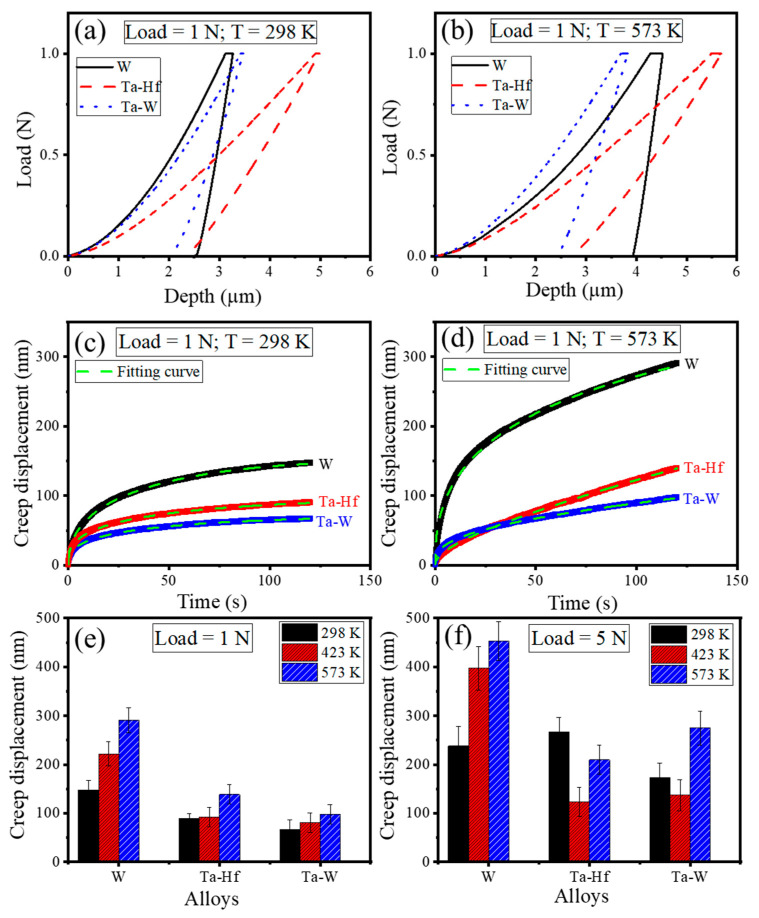
Nano-indentation load–displacement curves of W, HfTaTiVZr (Ta-Hf), and TaTiVWZr (Ta-W) alloys determined during creep experiments at (**a**) 1 N, 298 K and (**b**) 1 N, 573 K. Creep displacement versus holding time for all alloys at (**c**) 1 N, 298 K and (**d**) 1 N, 573. Creep displacement as a function of temperature for W, Ta-Hf and Ta-W alloys at (**e**) 1 N and (**f**) 5 N showing the increase of displacement with increasing temperature and load. Creep displacement was smaller for high entropy alloys compared to pure tungsten.

The representative load–displacement curves from the nano-indentation creep of the three systems are shown in Figure 2a,b at a load of 1 N and temperatures of 298 K and 573 K. The plots for other conditions were very similar and not shown here. An array of 2 × 6 indents were made with 100 μm separation covering several grains and therefore representative of several grain orientations. Overall, under the same load and temperature, the indentation depth in Ta-Hf HEA was greater than that in Ta-W and W, indicating the lowest hardness among the three systems studied. Figure 2c,d show the change of indentation depth versus holding time at the peak loads of 1 N (at 298 K and 573 K). There was an initial sharp rise in creep depth followed by slowing down the rate of increase. Creep displacement varied with the alloy composition, load, and temperature. Figure 2e,f represent the magnitude of total creep displacement as a function of temperature for W, Ta-Hf, and Ta-W alloys at 1 N and 5 N. The total creep displacement was in the range of 50 nm to 450 nm depending on alloy composition, holding load and temperature. A minimum of 12 indents were done in each condition for each alloy and the mean value was considered for comparison, the error bar likely attributed to the different crystal orientations. The creep displacement was larger at a higher temperature and load. Creep is a thermally activated process and partly depends on dislocation mobility, which increases at a higher temperature and load. The reduction in creep displacement for the two HEAs at 423 K may be explained in terms of dislocation mobility limitation by phonon-drag or secondary Peierls barrier [35]. The creep displacement for Ta-Hf and Ta-W high entropy alloys was roughly half of that of pure W, which may be attributed to the sluggish diffusion and highly distorted lattice structure in HEAs.

**Figure 3:**
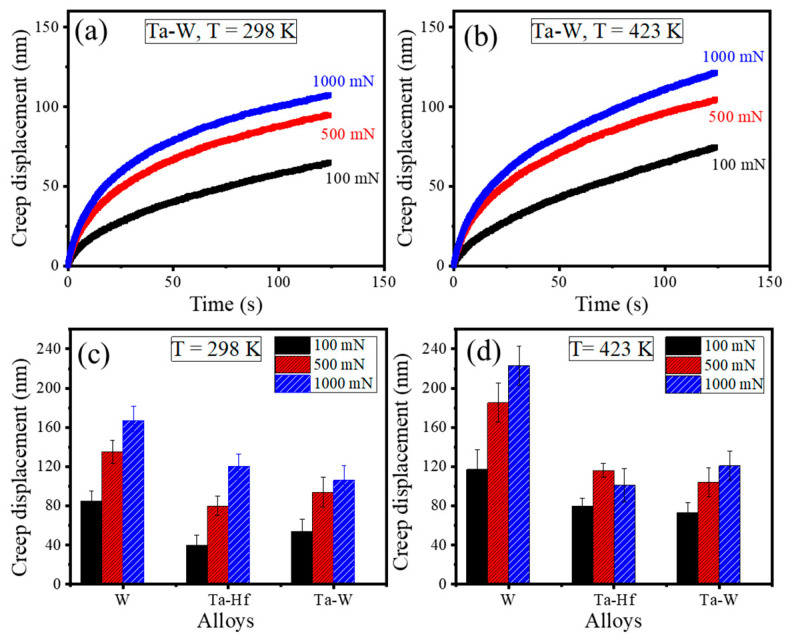
Creep displacement versus holding time for TaTiVWZr (Ta-W) alloy as a function of load at (**a**) 298 K and (**b**) 423 K. Maximum creep displacement dependence on applied load for W, Ta-Hf, and Ta-W at (**c**) 298 K and (**d**) 423 K showing larger creep displacement with increasing load and temperature.

The creep behavior of Ta-Hf and Ta-W HEAs was also studied under dynamic load and compared with W. The samples were loaded to predefined loads of 100 mN, 500 mN, and 1000 mN and held for 120 s under oscillatory loads. The amplitude of load was fixed at 10% of the peak load. Figure 3a,b show the creep displacement versus holding time as a function of load for Ta-W at 298 K and 423 K. The maximum creep depth increased with increasing load and same trend was observed for Ta-Hf and W. Figure 3c,d show the maximum creep displacement as a function of load during dwell time for all studied alloys at 298 K and 423 K, respectively. Increasing peak load and temperature led to an increase in creep displacement. Creep displacement was found to be similar for the two HEAs and lower than that of W in agreement with the static load data. The effect of surface oxidation was minimal (i.e., sample maintained shiny appearance) because of the Ar + H_2_ gas environment and high loads used for the creep tests.

## 4. Discussion

During holding in the constant loading stage, creep displacement (*h*) is a function of time (*t*) and expressed as [28]:

(1) $$h\left(t\right)=h_{0}+a{\left(t-t_{0}\right)}^{p}+kt$$

where $h_{0}$ and $t_{0}$ are indentation depth and time at the beginning of holding segment, and *a*, *p*, and *k* are fitting constants. Equation (1) showed a correlation coefficient *R*^2^ > 0.95 with the experimental data as shown in Figure 2c,d with dashed lines. For the self-similar Berkovich indentation tip, the following were used to obtain indentation strain rate (Equation (2)) and hardness (Equation (3)) [28]:

(2) $$\dot{\varepsilon }=\frac{1}{h}\frac{dh}{dt}$$

(3) $$H=\frac{P}{24.5h_{c}^{2}}$$

where $\frac{dh}{dt}$ is the time derivative of the fitted displacement–time curve (i.e., Equation (1)), *P* is the applied load, *h_c_* is contact depth, given by *h_c_* = *h_max_* −0.75 *P/S* for Berkovich indenter, and *h_max_* and S are maximum penetration depth and material stiffness, respectively. 

**Figure 4:**
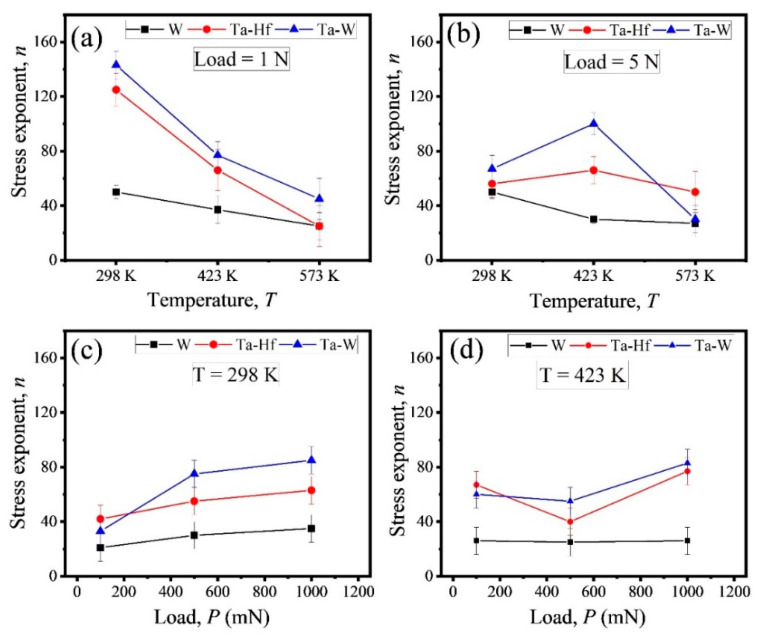
Stress exponent versus temperature for W, HfTaTiVZr (Ta-Hf), and TaTiVWZr (Ta-W) alloys at (**a**) 1 N and (**b**) 5 N showing the decrease of stress exponent with increasing temperature. Stress exponent versus load for all three systems at (**c**) 298 K and (**d**) 423 K showing indentation size effect of stress exponent.

The stress exponent (*n*), which provides valuable insight into creep deformation process, was calculated as [28]:

(4) $$n=\frac{1}{\frac{\partial \ln H}{\partial \ln\dot{\varepsilon }}}$$

where, $\frac{\partial \ln H}{\partial \ln\dot{\varepsilon }}$ is strain rate sensitivity. Figure 4a,b show the stress exponent for W, Ta-Hf, and Ta-W and its dependence on the temperature at static loads of 1 N and 5 N. The stress exponent as a function of dynamic loads for selected alloys at 298 K and 423 K are shown in Figure 4c,d. Each value of *n* was averaged for 12 independent indentations in the range of 20 to 140 depending on the alloy composition, temperature, and load. The creep stress exponent typically indicates the creep mechanism; *n* = 1 is associated with diffusion creep, *n* = 2 with grain boundary sliding, and *n* ≥ 3 with dislocation creep. Therefore, for the three current systems, despite the wide range of *n*, dislocation creep was the dominant mechanism. The stress exponent of HEAs at 1 N decreased significantly from 130-140 to 30-50 with an increase in temperature due to thermally activated dislocations at elevated temperature; however, the degree of reduction was not sharp in the case of pure W (from ~50 to ~30). The magnitude of stress exponent depends on the density of dislocations involved during deformation and whether the deformation is dominated by generation or annihilation of dislocations [36]. The decrease of *n* value with increasing temperature is due to the enhancement of dislocation movement and more thermal recovery at a higher temperature compared to their generation [17]. A similar trend was reported for other alloys [28,37]. The significantly larger drop in stress exponent with increasing temperature for HEAs may be due to less dislocation movement in their highly distorted lattice structure at room temperature leading to a high *n* value. With an increase in temperature, the thermally activated process and dislocation movement were more significant and stress exponent decreased sharply. Whereas, for W, even at room temperature, the annihilation rate may be high. At a load of 5 N, the same behavior was observed for W (i.e., decrease of *n* at a higher temperature); however, the two HEAs demonstrated slightly different behavior. There was an increase of *n* with increasing temperature from 298 K to 423 K and then reduction at 573 K. This is consistent with their lower creep displacement at an intermediate temperature which was shown in Figure 2f. The increase of stress exponent at 423 K for the high entropy alloys may be the result of the generation of more dislocations which act as a barrier for their movement [11]. Thermally activated cross slip of dislocations restricted their movement and resulted in an increase of *n* [38]. However, with a further increase in temperature to 573 K, thermally activated movement of dislocations was easier and caused a drop in *n*. This trend was seen only at the maximum load of 5 N, which may be due to a higher generation rate of dislocation at 5 N compared to 1 N [39], which increased the possibility of entanglement. Moreover, in BCC metals/alloys, the strain rate sensitivity and thermally activated deformation are controlled by kink-pair nucleation and screw dislocation propagation. The mobility of screw dislocations in BCC-HEAs may be restricted due to the lattice distortion. With increasing temperature, as a result of thermal fluctuations, the migration of a screw dislocation line from one Peierls valley to an adjacent one increases. In certain cases, upon propagation of dislocations in opposite directions under applied stress, their mobility is limited by phonon-drag or secondary Peierls barrier which results in higher stress exponent. In contrast, at a relatively higher temperature, the recombination of kinks with opposite line-directions may reduce the stress exponent [35]. The ISE on the creep stress exponent of BCC-HEAs was studied by nano-indentation using the DMA method. DMA was used to study the behavior of the alloys under oscillating loads and compared with the previous data at static loads. Figure 4c,d show the variation of *n* parameter as a function of load for Ta-Hf and Ta-W HEAs along with W in DMA tests. As peak load increased from 100 mN to 1000 mN (i.e., contact depth increased from 800 nm to 2500 nm), the *n* value increased for all samples at 298 K. This indicates an apparent size effect on the stress exponents, though all *n* values were in the range of dislocation dominated deformation mechanism. The indentation size effect for stress exponent has been reported in previous studies [18,24,40]. During the loading process, dislocations are generated in the plastic deformation zone beneath the indenter and their density is in direct proportion to the load/depth [19,39]. At low applied load, the dislocation generation rate may be slower than the dislocation annihilation rate, so lower stress exponent is obtained. At high load, the dislocation generation rate becomes very fast. During the holding time, the stress leads to dislocation propagation and they may interact and entangle with one another. Entanglement of dislocations may result in an increase of the stress exponent up to 100 at higher applied load or indentation depth [29,41]. At lower depth, the dislocations have higher mobility and diffusion rate because they are closer to the free surface, and as a result, the value of stress exponent is lower [42]. The mobility of screw dislocation in BCC metals (like Ta, Mo, and Fe) has been reported to be significantly enhanced near the free surface [39,42]. The indentation size effect of stress exponent was less significant at 423 K than at 298 K. This may be due to the counter balance of generation and annihilation rate of dislocations at elevated temperature. Less pronounced size effect for the hardness of materials at elevated temperatures has been also reported [43]. The value of *n* for the two HEAs at 423 K and the intermediate load of 500 mN was lower than that at 100 mN and 1000 mN. At 500 mN and elevated temperature, it is likely that the diffusion rate of dislocations was much higher than their generation rate which led to lower *n*; however, the mechanism remained unchanged.

The high value of stress exponent obtained using nano-indentation may be attributed to the complex stress state below the indenter [15,17,32,40]. The stress exponent is a strong function of the composition and microstructure of the alloy since mobile dislocation density and activation area may vary significantly [44]. The Ta-W HEA showed a higher *n* value in most of the conditions than Ta-Hf, which may be attributed to the dendrites in Ta-W which may act as barriers for dislocation movement. From Figure 4, it is evident that the stress exponent obtained during the DMA test is slightly lower than in the static test. This may be due to oscillatory load which may result in better dislocation propagation and a lower *n* value.

The activation volume (V*), which depends on stress exponent and hardness (*H*), was calculated for further insight into the creep mechanism as [17]:

(5) $$V^{*}=\frac{3\cdot \sqrt{3}\cdot k\cdot T\cdot n}{H}$$

where *k* is Boltzmann constant and *T* is temperature. The average hardness value over the holding stage at each temperature was used to calculate the activation volume. The averaged activation volume was 0.25 ± 0.1 nm^3^ (~13*b*^3^), 0.5 ± 0.11 nm^3^ (~20*b*^3^) and 0.4 ± 0.09 nm^3^ (~16*b*^3^) for W, Ta-Hf, and Ta-W, respectively. Lattice parameter (*a*_0_) is ~0.34 nm for current HEAs [9] and ~0.31 nm for W, and *b* = 1/2 *a*_0_ [111] is Burgers vector of BCC alloys/metals. The activation volumes for all three systems were in the range for kink-pair nucleation and movement of screw dislocations in BCC alloys/metals [35]. V* value is in the range of 10*b*^3^–1000*b*^3^ for dislocation creep while diffusion-mediated creep is typically associated with lower values of V*. Tungsten showed a slightly smaller activation volume compared to the two HEAs. Activation volume describes the degree of dislocation nucleation, the smaller activation volume indicating easier nucleation of dislocations [35]. Activation volume for BCC CoCrFeNiCuAl_2.5_ thin film HEA measured using a Berkovich nano-indenter was reported to be ~0.5 nm^3^ [15], while FCC CoCrFeCuNi thin film and coarse-grained CoCrFeMnNi showed one order of magnitude lower activation volume of 0.08 nm^3^ and 0.05 nm^3^, respectively [15,27].

**Figure 5:**
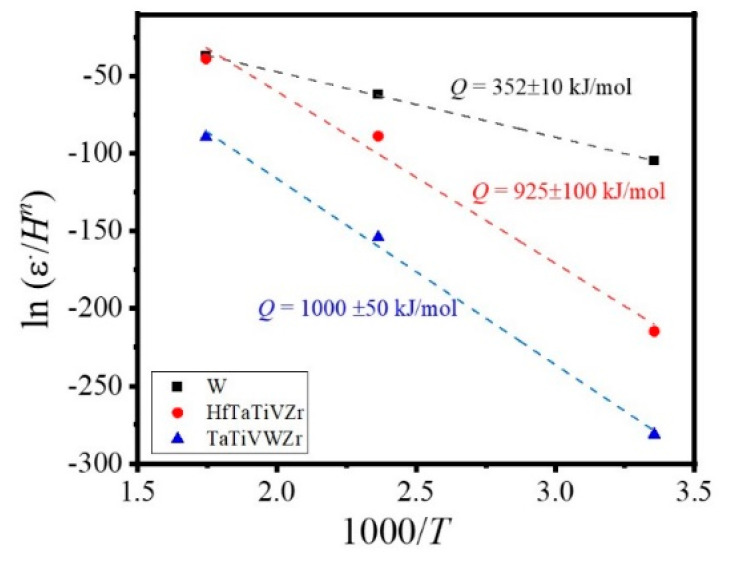
ln($\dot{\varepsilon }$/*H^n^*) versus 1000/*T* with slope giving the activation energy (*Q*) for W, HfTaTiVZr, and TaTiVWZr high entropy alloys. The activation energies for the current refractory high entropy alloys were higher than tungsten by almost a factor of three.

The temperature dependence of indentation creep rate is empirically correlated through a power-law relation [45]:

(6) $$\dot{\varepsilon }=A\sigma^{n}\exp\left(-\frac{Q}{RT}\right)\approx AH^{n}\exp\left(-\frac{Q}{RT}\right)$$

where *A* and *R* are structure-dependent and universal gas constant, respectively, and *Q* is the activation energy. A plot of ln($\dot{\varepsilon }$/*H^n^*) versus 1/*T* yields a slope of −*Q/R* [46,47] as plotted in Figure 5 for all the studied alloys. The average strain rate and hardness over holding time at each temperature were selected for analysis. Linear regression indicated creep activation energy of 352 ± 10 kJ/mol, 925 ± 100 kJ/mol, and 1000 ± 50 kJ/mol for W, Ta-Hf, and Ta-W, respectively. The calculated higher activation energy of 900–1000 kJ/mol for HEAs compared to pure W may be associated with severe lattice distortion and sluggish diffusion in HEAs resulting in a greater degree of dislocation interaction and supporting their higher creep resistance [48]. Activation energy for CoCrFeNiMn [12] and precipitation-hardened (FeCoNiCr)_94_Ti_2_Al_4_ [49] HEAs were reported to be ~300–400 kJ/mol and ~300–800 kJ/mol, respectively, from tensile tests. However, to the best of the authors’ knowledge, there are no reports on activation energy of HEAs by the nano-indentation creep test. In summary, the high activation energy for Ta-Hf and Ta-W compared to pure refractory metals like tungsten support their excellent creep resistance. This suggests the potential use of these alloys in next-generation nuclear reactors as well as fossil fuel power plants where refractory metals are currently used.

## 5. Conclusions

In summary, indentation creep tests for reduced activity HfTaTiVZr and TaTiVWZr HEAs were performed by static and dynamic loads at 298 K, 423 K and 573 K. The creep mechanism of the alloys were compared in terms of stress exponent and activation volume. Comparison of the creep resistance of the three systems was done based on creep displacement and activation energy. The following conclusions were drawn:

(1) The creep exponent was in the range of 20–140 and activation volume was in the range of 13–20*b*^3^, indicating that the time-dependent deformations for all alloys were dislocation dominated.
(2) The stress exponent decreased with increasing temperature owing to thermally activated dislocations and the reduction was sharper for HEAs compared to pure W.
(3) The creep exponent increased with increasing load (depth) leading to an apparent size effect due to a higher generation rate of dislocation and their entanglement at larger penetration depth. A higher diffusion/annihilation rate of dislocations near the free surface at a smaller depth may be another possible explanation.
(4) HEAs showed smaller creep displacement and higher activation energy compared to pure tungsten, which may be attributed to sluggish diffusion and severe lattice strains.
